# Short structural variants as informative genetic markers for ALS disease risk and progression

**DOI:** 10.1186/s12916-021-02206-y

**Published:** 2022-01-17

**Authors:** Frances Theunissen, Loren L. Flynn, Ryan S. Anderton, P. Anthony Akkari

**Affiliations:** 1 Perron Institute for Neurological and Translational Science, First floor, RR block, QEII Medical Centre, 8 Verdun St, Nedlands, WA 6009 Australia; 2 Centre for Neuromuscular and Neurological Disorders, University of Western Australia, Nedlands, WA Australia; 3 Centre for Molecular Medicine and Innovative Therapeutics, Murdoch University, Perth, WA Australia; 4 Black Swan Pharmaceuticals, Wake Forrest, NC USA; 5 Faculty of Medicine, Nursing, Midwifery and Health Sciences, University of Notre Dame Australia, Fremantle, WA 6160 Australia; 6 Division of Neurology, Duke University Medical Centre, Duke University, Durham, NC USA

**Keywords:** Genetic marker, Structural variant, Amyotrophic lateral sclerosis, Clinical trials, Participant selection, Enrichment tool, Responder sub-population

## Abstract

There is considerable variability in disease progression for patients with amyotrophic lateral sclerosis (ALS) including the age of disease onset, site of disease onset, and survival time. There is growing evidence that short structural variations (SSVs) residing in frequently overlooked genomic regions can contribute to complex disease mechanisms and can explain, in part, the phenotypic variability in ALS patients. Here, we discuss SSVs recently characterized by our laboratory and how these discoveries integrate into the current literature on ALS, particularly in the context of application to future clinical trials. These markers may help to identify and differentiate patients for clinical trials that have a similar ALS disease mechanism(s), thereby reducing the impact of participant heterogeneity. As evidence accumulates for the genetic markers discovered in *SQSTM1*, *SCAF4*, and *STMN2*, we hope to improve the outcomes of future ALS clinical trials.

## Background

Many key cellular processes are known to be disrupted in amyotrophic lateral sclerosis (ALS) such as RNA metabolism, mitochondrial dysfunction, oxidative stress, protein aggregation, impaired axonal transport, and cytoskeletal dysfunction, reviewed in [1]. Variation in the expression of genes involved in these processes may increase disease risk and/or influence the rate of disease progression [2–4]. At present, there is a lack of genetic markers for the different ALS disease subtypes, as well as a lack of genetic indicators of disease risk and/or trajectory. The heterogeneous clinical presentation and diverse rates of progression makes identifying ALS patients with similar disease mechanisms extremely challenging, and undoubtedly contributes to clinical trial failures [5].

Establishing molecular targets and genetic markers for ALS can lead to improved patient stratification for clinical trials and might enable positive treatments to be identified for specific patient subgroups. Van Eijk and Eijkemans [6] recently demonstrated that genotypic data for unc-13 homolog a (*UNC13A*), myelin-associated oligodendrocyte basic protein (*MOBP*), and the repeat expansion in c9orf720-SMCR8 complex subunit (*C9orf72*) could influence both primary and secondary outcomes including survival, ALS functional rating scale (ALFSRS) and forced vital capacity (FVC) measures. Additionally, a retrospective meta-analysis of three lithium carbonate clinical trials revealed that contrary to the reported negative outcomes, patients with the *UNC13A* (C/C) genotype had actually responded to the lithium carbonate [7]. This study provides evidence that genetic markers can inform clinical trial outcomes and should be incorporated into clinical trial design. Intuitively, genetic regions that are highly variable, known as structural variants (SVs), will likely be more informative as genetic markers than single nucleotide polymorphisms (SNP), due to a larger number of potential genotypes [8]. These variable regions of the genome have been predominantly under-characterized [9, 10]; however, the scientific community is beginning to appreciate the need to investigate polymorphic loci as potential disease modifying regions.

Structural variants (SVs) have typically been defined as insertions, deletions, inversions, and microsatellites that can be repeated hundreds of times within the genome that are greater than 1 kb in length [11]. Short structural variants (SSVs) have been predominately overlooked in the context of ALS and encompass that same class of variants as the classical definition (e.g., short tandem repeat, microsatellite, insertion/deletion, inversion, polynucleotide repeat) but are much shorter in size, typically < 50 bp in length [12]. Approximately 4 million SSVs exist within the human genome and have been previously described [13, 14]. It is possible that some of the “missing heritability” in ALS could be explained by more common SSVs with small effect sizes that have not yet been identified [13, 15]. Importantly, changes in the size and composition of both SV/SSVs can have a significant impact on the binding of regulatory elements that modulate RNA processing and gene expression [16]. SSVs have been implicated in many complex diseases, including ALS and other neurodegenerative diseases [17], such as Parkinson’s and Alzheimer’s disease [18–20]. The ability of SSVs to alter gene expression is dependent on their location within and around the gene or intergenic region, with their effects occurring via several mechanisms including the following: influencing the binding of regulatory elements, mRNA splicing and processing, genome folding and higher order structure, and translation. This may differentiate mechanisms of disease pathogenesis, including risk of disease, risk for a specific phenotype, symptom presentation, disease course, and response to treatment, between individuals [21].

Recent in silico mapping of known ALS-linked genes has predicted a number of as yet unresolved short tandem repeats within each of these genes, that are likely polymorphic, and could influence gene expression and contribute to disease risk for ALS [15, 22]. Importantly, in the most recent ALS genome wide association study (GWAS) conducted by Van Rheenen and Van der Spek [23], 15 risk loci were identified, with 8 loci previously reported in GWAS studies [24–26]. This GWAS study was unique; in addition to screening pathogenic rare burden variants, it also incorporated short tandem repeats, RNA-sequencing, and methylation datasets to prioritize causal genes within identified ALS risk loci. Of note, a polymorphic tetra-nucleotide repeat downstream of ALS-linked gene NIMA-related kinase 1 (*NEK1*) was reported to be associated with increased ALS disease risk. The *NEK1* SSV was in linkage disequilibrium with the top hit *NEK1* SNP reported in this study and may help to further explain phenotypic variability and disease penetrance between patients carrying different *NEK1* mutations, particularly since the SNP alone could not reliably determine its contribution to ALS risk [23]. Furthermore, another recent study has investigated SVs in 6580 whole genome sequences (4315 ALS and 1880 controls) from the Project MinE cohort to determine genotype-phenotype correlations in known ALS genes. Al Khleifat, Iacoangeli [27] reported that structural variants in *C9orf72* (repeat expansion), Valosin containing protein *VCP* (inversion) and erb-b2 receptor tyrosine kinase 4, *ERBB4* (deletion) are variously associated with ALS disease risk and phenotype. The variant caller used in this study detected multiple classes of SVs, however, small inversions and insertion/deletions < 200 bp could not be analyzed by the Manta platform [27]. Further investigation into variants outside of the know ALS genes in addition to wet lab validation is required to gain a holistic understanding of the contribution of genetic variation to ALS risk and phenotype [27].

In addition to contributing to disease risk, SSVs can also be an informative tool for clinical trial participant selection, as demonstrated in Alzheimer’s disease, in the case of translocase of outer mitochondrial membrane 40 (*TOMM40*) [28]. Although the age-of-onset distributions for Alzheimer’s disease have been well-established since 1993 [29], the basis of this distribution was only partially explained by the apolipoprotein E (*APOE*) genotype, suggesting that other genetic factors must contribute to age-of-onset for the disease [30]. In fact, the combination of the *APOE* genotype alongside the SSV genotype, a poly-T repeat in *TOMM40*, was subsequently shown to account for > 98% of the clinical age-of-onset distributions in the Caucasian population [20, 28]. Combining these genotypes allowed the generation of the clinical age-of-onset risk algorithm [31] to be developed to inform participant selection for the TOMMORROW clinical trial [32], with selection based on the genotype of individuals and their corresponding risk of disease/age-of-onset prediction. Evidently, this finding demonstrates that informative SSVs can be used as an enrichment tool for clinical trials, thus informing participant selection.

## Innovative approach for SSV discovery

In ALS, the clinical presentation can manifest differently in family members sharing the same ALS-linked variant (incomplete penetrance), suggesting multiple factors, including both genetic and non-genetic factors, can contribute to the progression of the disease [15, 21, 33]. With this in mind, we have used an established structural variant evaluation system (dbSSV) [22] to identify candidate SSVs within and around confirmed ALS loci [15]. Using a systematic approach to candidate GWAS loci, analysis of SSVs in these genomic regions will likely identify common variants that subtly influence gene function, in some cases leading to ALS. The dbSSV software [22] focuses specifically on identifying SSVs and scores them against 24 different properties, including data describing the location and definition of the variation, variability indicators, repeat context, gene context, transcription factor and microRNA binding sites, other regulatory markers, conservation, position within a linkage disequilibrium block, GWAS signals, and tissue-specific regulatory signals. Based on these scores, a short list of SSVs is generated with each total score suggesting the likelihood of the variant having significant biological effects and contributing to disease risk. Using this method, we have now identified and published several novel genetic markers, discussed below. Investigating SSVs in ALS-linked genes will help to better understand differences between individual patient phenotypes and disease progression.

## Insertion/deletion in *SQSTM1* is associated with disease in familial *SOD1* patients

Sequestosome 1 (*SQSTM1*) encodes P62, an adaptor protein that binds ubiquitylated protein aggregates and delivers them to the autophagosome for degradation. With an essential role in protein clearance, it is not surprising that P62 dysfunction is implicated in neurodegenerative diseases that are governed by abnormal protein inclusions. Thus, SSVs within *SQSTM1* may contribute to the diverse presentations observed between ALS patients by influencing P62 expression and autophagic clearance. In our recently published association study of a North American cohort of familial ALS (fALS) and sporadic (sALS) patients (*n* = 403) and age matched controls (*n* = 562), a small cytosine/adenine (CAAA) insertion deletion (I/D) was associated with fALS, particularly in familial superoxide dismutase 1 (*SOD1*) mutation positive patients, but not with sALS patients [34]. Furthermore, the presence of the insertion variant appears to translate to a stepwise decrease in *SQSTM1* expression in healthy olfactory neurosphere-derived cells, with the I/I genotype resulting in a 2.5-fold reduction in transcript levels [34]. The observations of the insertion/deletion influencing *SQSTM1* expression has previously been reported by an independent study in a screen of 17 different healthy tissue types [35]. By this weight of evidence, the CAAA variant needs to be further examined as a contributor to ALS disease mechanisms, particularly since *SQSTM1* plays a critical role in autophagy, and mutations in this gene can be a direct cause of ALS and other neurodegenerative diseases [36, 37]. Future therapeutic studies to modulate autophagy should take into consideration the potential impact of this SSV on P62 expression.

## Poly-T repeat in *SCAF4* is a genetic marker for disease risk and survival in familial ALS

Studies of common *SOD1*-ALS mutations have suggested that a disease modifying factor located nearby *SOD1* may influence the penetrance of *SOD1* p.D91A (D90A) and p.A5V (AV4) mutations (historical names noted in brackets are not reflective of the amino acid position), thus contributing broadly to ALS risk [38, 39]. Our laboratory reported an 11-18 thymine repeat, located within the 3′UTR of the neighboring gene, SR-related CTD associated factor 4 (*SCAF4*) [40]. The function of *SCAF4* has recently been elucidated, with an essential role in RNA processing through regulating transcript elongation [41]. Variants in *SCAF4* have been reported to cause impaired RNA processing and can result in neurodevelopmental disorders [42]. Interestingly, the poly-T variant in *SCAF4* is flanked by two binding sites for transcription factor RNA polymerase II subunit A (POLR2A); therefore, changes in the length of this variant may influence the binding of POLR2A, thus affecting transcription of nearby genes (*SOD1*) and may therefore play a role in ALS related neurodegeneration. A case control association study in fALS patients (*n* = 190) and healthy age matched controls (*n* = 560) revealed that the 18 T repeat is associated with ALS risk for the entire cohort, including those without *SOD1*-linked mutations (*n* = 27). The 18 T allele was also associated with a 26 month reduction in survival time but was not associated with age at disease onset [40]. Future studies should investigate the functional impact of this variant and determine whether thymine repeat length influences transcript elongation of genes that are regulated by *SCAF4*. Additionally, this SSV may also help to identify patients that could benefit from a *SOD1* targeted therapy, such as the SOD1 suppressing antisense therapy Tofersen (currently in phase III extension study) [43].

## Short tandem repeat in *STMN2* is associated with sporadic ALS disease risk and clinical phenotype

The microtubule regulator stathmin-2 (*STMN2*) was recently identified as a gene with strong therapeutic potential for ALS [44, 45]. *STMN2* is involved in axonal grown and repair and is directly regulated by the ALS-linked gene TAR DNA-binding protein 43 (*TARDBP*). When *TARDBP* protein TDP-43 is depleted or mislocalized, as occurs in ALS, STMN2 protein is also depleted [44, 45]. To understand the implications of this TDP- 43:STMN2 relationship and ALS phenotype, it is essential to characterize the natural variation in *STMN2* expression.

We recently examined the *STMN2* gene for candidate SSVs that may affect the binding of regulatory elements and, therefore, influence gene expression. We identified a variable length cytosine/adenine (CA) short tandem repeat within *STMN2* in a cohort of Caucasian sALS patients (*n* = 321) and healthy age matched controls (*n* = 332). We have identified the first genetic association with *STMN2* and both sALS disease risk and age-at-disease onset [46]. In 143 patients where end-point survival data was available, when categorized according to site of disease onset (bulbar vs spinal), the bulbar cases carrying the risk genotype had significantly shorter survival times than other bulbar cases. Moreover, this effect on survival was not abrogated when controlling for sex or age. Furthermore, in an Australian longitudinal sALS cohort (*n* = 67), ALSFRS scores were significantly lower in patients carrying the risk genotype. Following these clinical associations, stathmin-2 mRNA expression was shown to be reduced in sALS patient olfactory neurosphere-derived cells. When accounting for CA genotype, a trend for reduced expression of stathmin-2 mRNA was also observed in sALS patients and control laser-captured spinal motor neurons [46].

This work points to a novel mechanism by which this SSV may regulate *STMN2* gene expression and could further explain the recently elucidated *STMN2* cryptic exon mechanism [44, 45] and its influence on neurodegenerative disease phenotype [47, 48]. The genetic validation of *STMN2* significantly adds to the weight of evidence that this gene is important in ALS [46]. The *STMN2* genetic marker may therefore be a useful tool for cohort selection in clinical trials or to stratify patient response. Further, this discovery has broad implications for clinical assessment and therapeutic development and should be incorporated in future clinical trials targeting this gene.

## Application of SSVs to clinical trials

Recently, the Treatment Research Initiative to Cure ALS (TRICALS) has brought the urgent need to reform ALS clinical trial design to the forefront of the literature [5, 49]. In particular, the major concerns raised are related to the stringent patient selection criteria and analytical strategy of phase 3 clinical trials. Van Eijk and Nikolakopoulos [49] highlight the use of patient risk profiles as a strategy to provide more informative selection criteria that will help to improved randomization, enable risk-based subgroup analyses, and increase the statistical power of clinical trials. Patient risk profiles are based on a multivariate analysis of several patient characteristics (i.e., age of onset, site of onset, vital capacity, diagnostic delay, ALSFRS etc.), creating a “prognostic summary” for each patient [49]. This is likely to help increase the number of eligible patients for trials, while reducing patient drop out and exclusion rates, thus increasing the generalizability of the trial [49]. The authors noted that they did not include *C9orf72* repeat expansion as a factor in their predictive model, as it did not significantly improve the predictive power of the model and may pose additional logistical constraints (i.e., genotyping and counseling patients) prior to clinical trial randomization [49]. However, one must consider that although the *C9orf72* repeat expansion is applicable to 40% of fALS patients and 6% sALS patients [50], incomplete penetrance is likely to be the complicating factor that influences the predictive capacity of this variant. Incorporating genetic variants into the prognostic summary may not improve prognostic predictions, since the clinical characteristics that the variant may be associated with are already individually incorporated into the model. The true value of patient genotype information is in balancing the clinical trial treatment arms, allowing sub-group analyses for patients that may have similar underlying disease mechanisms or ALS risk factors. It is likely that the eligibility window for patient risk profiles will need evaluation on a study-by-study basis, particularly since this will be influenced by the type of trial, i.e., broad vs genotype targeted treatment [49]. However, the use of patient genotype information will be pertinent to maximizing the information gained from a prospective clinical trial on potential responders. Across the most frequently studied indications, it has been shown that genetically validated targets are twice as likely to succeed in clinical development, transitioning from phase 1 to approval [51]. Therefore, the use of association studies to identify ALS genetic markers will help to guide selection of appropriate drug targets for sub-populations and help lower the rate of failure in clinical development programs.

Tofersen is currently the most promising disease modifying therapeutic for ALS and recently completed phase III (NCT02623699) with its long-term extension study currently postponed (NCT03070119) [43]. Tofersen was initially designed as a genetically targeted therapeutic for *SOD1* mutation positive patients [43]; however, evidence is accumulating that this therapy may also benefit other forms of sALS, where SOD1 misfolding and aggregation is also a pathological feature [52, 53]. The use of genetic information in identifying the target population (mutation positive patients) in its initial early trial stage was integral to the phase I/II trial success [43]. Incorporation of genetic information in early-stage clinical development allowed initial efficacy to be shown and can be built upon in the hope of treating a broader patient population. Genetic variants that are associated with SOD1 aggregation within sALS cohorts will be necessary to identify those sALS patients who are likely to respond to a SOD1 targeting therapy. With the evolution of personalized treatment approaches using antisense oligonucleotides for neurodegenerative disorders [54], there is an absolute need to incorporate informative SSV genetic markers into early stage drug development. This will help to identify patients that have similar ALS disease mechanisms that would benefit from a targeted antisense therapy.

## Conclusion

Currently, there is no effective treatment for ALS, and over the past 20 years, more than 60 controlled trials of putative ALS therapeutics have failed to demonstrate clinical efficacy [55, 56]. Current treatment options are non-specific and only extend survival by ~3 months in some patients, and furthermore, at present, there is no method to determine which patients are more likely to respond to a particular ALS therapeutic.

The continued discovery and evaluation of novel SSVs will undoubtedly shed light on the pathogenic mechanisms of ALS neurodegeneration. At present, there are few biomarkers/genetic markers that allow patient stratification according to disease mechanism [57–60] and treatment efficacy can only be evaluated by clinical measures during current clinical trials [34, 38]. With increasing evidence from our laboratory that SSVs do contribute to ALS risk and have disease-modifying effects [15, 34, 40, 46], investigations need to incorporate SSVs into genetic studies and clinical trial design [5]. There is an urgent need to establish well-characterized genetic markers that can be used to inform on the validity of certain treatment approaches. As ALS is a complex and heterogeneous disorder, with a varied clinical phenotype and disease trajectory, personalized medicine approaches will be more likely to result in successful treatments because they can directly target the underlying disease mechanism [54]. Therefore, it is crucial to be able to identify patient subgroups and develop compounds that are more likely to be effective in genetically defined subgroups of patients, thus reducing the impact of participant heterogeneity.

The authors recognize the challenges we face in current ALS clinical trials. Moving forward, we must examine the potential of these SSVs as a tool for patient stratification in retrospective clinical trials cohorts. This will result in the accelerated development of these genetic markers, fast tracking them into current and future clinical trials. Undoubtedly, as the data accumulates for these genetic markers, we are hopeful this will translate into identifying responder populations of ALS patients, allowing drug development to continue for specific subsets of patients. This is likely to significantly change the way clinical trials are conducted in ALS moving forward.

## Data Availability

Not applicable.

## Abbreviations

ALS: Amyotrophic lateral sclerosis
SVs: Structural variants
SSVs: Short structural variants
*UNC13A*: Unc-13 homolog a
*MOBP*: Myelin-associated oligodendrocyte basic protein
*C9orf72*: C9orf720-SMCR8 complex subunit
ALFSRS: ALS functional rating scale
FVC: Forced vital capacity
SNP: Single nucleotide polymorphism
GWAS: Genome wide association study
*NEK1*: NIMA-related kinase 1
*TOMM40*: Translocase of outer mitochondrial membrane 40
*APOE*: Apolipoprotein E
dbSSV: Structural variant evaluation system
*SQSTM1*: Sequestosome 1
fALS: Familial ALS
sALS: Sporadic ALS
*SOD1*: Superoxide dismutase 1
I/D: Insertion deletion
*SCAF4*: SR-related CTD associated factor 4
POLR2A: RNA polymerase II subunit A
*STMN2*: Stathmin-2
*TARDBP*: TAR DNA-binding protein 43
TRICALS: Treatment Research Initiative to Cure ALS

## References

[1] Mejzini R, Flynn LL, Pitout IL, Fletcher S, Wilton SD, Akkari PA (2019). ALS genetics, mechanisms, and therapeutics: where are we now?. Front Neurosci.

[2] D’Erchia AM, Gallo A, Manzari C, Raho S, Horner DS, Chiara M, Valletti A, Aiello I, Mastropasqua F, Ciaccia L, Locatelli F, Pisani F, Nicchia GP, Svelto M, Pesole G, Picardi E (2017). Massive transcriptome sequencing of human spinal cord tissues provides new insights into motor neuron degeneration in ALS. Sci Rep.

[3] Krokidis MG, Vlamos P (2018). Transcriptomics in amyotrophic lateral sclerosis. Front Biosci (Elite Ed).

[4] Recabarren-Leiva D, Alarcón M (2018). New insights into the gene expression associated to amyotrophic lateral sclerosis. Life Sci.

[5] Van Eijk RPA, Kliest T, McDermott CJ, Roes KCB, Van Damme P, Chio A (2020). TRICALS: creating a highway toward a cure. Amyotroph Lateral Scler and Frontotemporal Degener.

[6] Van Eijk RP, Eijkemans MJ, Nikolakopoulos S, Jansen MD, Westeneng H-J, Van Eijk KR, et al. Pharmacogenetic interactions in amyotrophic lateral sclerosis: a step closer to a cure? Pharmacogenomics J. 2019:1–7. 10.1038/s41397-019-0111-3 31624333

[7] Van Eijk RP, Jones AR, Sproviero W, Shatunov A, Shaw PJ, Leigh PN (2017). Meta-analysis of pharmacogenetic interactions in amyotrophic lateral sclerosis clinical trials. Neurol.

[8] Roses A (2016). Polyallelic structural variants can provide accurate, highly informative genetic markers focused on diagnosis and therapeutic targets: Accuracy vs. Precision Clin Pharmacol Ther.

[9] Cameron DL, Di Stefano L, Papenfuss AT (2019). Comprehensive evaluation and characterisation of short read general-purpose structural variant calling software. Nat Commun.

[10] Ebbert MTW, Jensen TD, Jansen-West K, Sens JP, Reddy JS, Ridge PG, Kauwe JSK, Belzil V, Pregent L, Carrasquillo MM, Keene D, Larson E, Crane P, Asmann YW, Ertekin-Taner N, Younkin SG, Ross OA, Rademakers R, Petrucelli L, Fryer JD (2019). Systematic analysis of dark and camouflaged genes reveals disease-relevant genes hiding in plain sight. Genome Biol.

[11] Conrad DF, Hurles ME (2007). The population genetics of structural variation. Nat Genet.

[12] Mahmoud M, Gobet N, Cruz-Dávalos DI, Mounier N, Dessimoz C, Sedlazeck FJ (2019). Structural variant calling: the long and the short of it. Genome Biol.

[13] Frazer KA, Murray SS, Schork NJ, Topol EJ (2009). Human genetic variation and its contribution to complex traits. Nat Rev Genet.

[14] Auton A, Abecasis GR, Altshuler DM, Durbin RM, Abecasis GR, Bentley DR (2015). A global reference for human genetic variation. Nature.

[15] Theunissen F, Flynn LL, Anderton RS, Mastaglia F, Pytte J, Jiang L (2020). Structural variants may be a source of missing heritability in sALS. Front Neurosci.

[16] Chiang C, Scott AJ, Davis JR, Tsang EK, Li X, Kim Y (2017). The impact of structural variation on human gene expression. Nat Genet.

[17] Beck J, Poulter M, Hensman D, Rohrer JD, Mahoney CJ, Adamson G, Campbell T, Uphill J, Borg A, Fratta P, Orrell RW, Malaspina A, Rowe J, Brown J, Hodges J, Sidle K, Polke JM, Houlden H, Schott JM, Fox NC, Rossor MN, Tabrizi SJ, Isaacs AM, Hardy J, Warren JD, Collinge J, Mead S (2013). Large C9orf72 hexanucleotide repeat expansions are seen in multiple neurodegenerative syndromes and are more frequent than expected in the UK population. Am J Hum Genet.

[18] Bakeberg MC, Hoes ME, Gorecki AM, Theunissen F, Pfaff AL, Kenna JE, Plunkett K, Kõks S, Akkari PA, Mastaglia FL, Anderton RS (2021). The TOMM40 ‘523’polymorphism in disease risk and age of symptom onset in two independent cohorts of Parkinson’s disease. Sci Rep.

[19] Bakeberg MC, Gorecki AM, Pfaff AL, Hoes ME, Kõks S, Akkari PA, Mastaglia FL, Anderton RS (2021). TOMM40 ‘523’ poly-T repeat length is a determinant of longitudinal cognitive decline in Parkinson’s disease. NPJ Parkinson’s Dis.

[20] Roses A, Lutz M, Amrine-Madsen H, Saunders A, Crenshaw D, Sundseth S (2010). A TOMM40 variable-length polymorphism predicts the age of late-onset Alzheimer’s disease. Pharmacogenomics J.

[21] Shatunov A, Al-Chalabi A (2021). The genetic architecture of ALS. Neurobiol Dis.

[22] Saul R, Lutz MW, Burns DK, Roses AD, Chiba-Falek O (2016). The SSV evaluation system: a tool to prioritize short structural variants for studies of possible regulatory and causal variants. Hum Mutat.

[23] Van Rheenen W, Van der Spek RAA, Bakker MK, JJFAv V, Hop PJ, RAJ Z, et al. Common and rare variant association analyses in amyotrophic lateral sclerosis identify 15 risk loci with distinct genetic architectures and neuron-specific biology. Nat Genet. 2021;53(12):1636–48. 10.1038/s41588-021-00973-1 PMC8648564 34873335

[24] Van Rheenen W, Shatunov A, Dekker AM, McLaughlin RL, Diekstra FP, Pulit SL (2016). Genome-wide association analyses identify new risk variants and the genetic architecture of amyotrophic lateral sclerosis. Nat Genet.

[25] Benyamin B, He J, Zhao Q, Gratten J, Garton F, Leo PJ, Liu Z, Mangelsdorf M, al-Chalabi A, Anderson L, Butler TJ, Chen L, Chen XD, Cremin K, Deng HW, Devine M, Edson J, Fifita JA, Furlong S, Han YY, Harris J, Henders AK, Jeffree RL, Jin ZB, Li Z, Li T, Li M, Lin Y, Liu X, Marshall M, McCann EP, Mowry BJ, Ngo ST, Pamphlett R, Ran S, Reutens DC, Rowe DB, Sachdev P, Shah S, Song S, Tan LJ, Tang L, van den Berg LH, van Rheenen W, Veldink JH, Wallace RH, Wheeler L, Williams KL, Wu J, Wu X, Yang J, Yue W, Zhang ZH, Zhang D, Noakes PG, Blair IP, Henderson RD, McCombe PA, Visscher PM, Xu H, Bartlett PF, Brown MA, Wray NR, Fan D (2017). Cross-ethnic meta-analysis identifies association of the GPX3-TNIP1 locus with amyotrophic lateral sclerosis. Nat Commun.

[26] Nicolas A, Kenna KP, Renton AE, Ticozzi N, Faghri F, Chia R (2018). Genome-wide analyses identify KIF5A as a novel ALS gene. Neuron.

[27] Al Khleifat A, Iacoangeli A, JJfa VV, Bowles H, RAJ Z, Moisse M, et al. Structural variation analysis of 6,500 whole genome sequences in amyotrophic lateral sclerosis. NPJ Genom Med. 2021. https://kclpure.kcl.ac.uk/portal/en/publications/structural-variation-analysis-of-6500-whole-genome-sequences-inamyotrophic-lateral-sclerosis(c59ada0f-35ff-4d57-a26c-16ac3e6720f4).html. 10.1038/s41525-021-00267-9 PMC8799638 35091648

[28] Roses AD, Akkari PA, Chiba-Falek O, Lutz MW, Gottschalk WK, Saunders AM, Saul B, Sundseth S, Burns D (2016). Structural variants can be more informative for disease diagnostics, prognostics and translation than current SNP mapping and exon sequencing. Expert Opin Drug Metab Toxicol.

[29] Corder EH, Saunders AM, Strittmatter WJ, Schmechel DE, Gaskell PC, Small G (1993). Gene dose of apolipoprotein E type 4 allele and the risk of Alzheimer’s disease in late onset families. Science.

[30] Raybould R, Sims R (2021). Searching the dark genome for Alzheimer’s disease risk variants. Brain Sci.

[31] Crenshaw DG, Gottschalk WK, Lutz MW, Grossman I, Saunders AM, Burke JR, Welsh-Bohmer KA, Brannan SK, Burns DK, Roses AD (2013). Using genetics to enable studies on the prevention of Alzheimer’s disease. Clin Pharmacol Ther.

[32] Burns DK, Chiang C, Welsh-Bohmer KA, Brannan SK, Culp M, O'Neil J, Runyan G, Harrigan P, Plassman BL, Lutz M, Lai E, Haneline S, Yarnall D, Yarbrough D, Metz C, Ponduru S, Sundseth S, Saunders AM, TOMMORROW Study Investigators (2019). The TOMMORROW study: design of an Alzheimer’s disease delay-of-onset clinical trial. Alzheimer’s Dement: Transl Res Clin Interv.

[33] Al-Chalabi A, Calvo A, Chio A, Colville S, Ellis CM, Hardiman O (2014). Analysis of amyotrophic lateral sclerosis as a multistep process: a population-based modelling study. Lancet Neurol.

[34] Pytte J, Anderton RS, Flynn LL, Theunissen F, Jiang L, Pitout I, et al. Association of a structural variant within the SQSTM1 gene with amyotrophic lateral sclerosis. Neurol Genet. 2020;6:e406. 10.1212/NXG.0000000000000406. 10.1212/NXG.0000000000000406 PMC7061286 32185242

[35] Fotsing SF, Margoliash J, Wang C, Saini S, Yanicky R, Shleizer-Burko S, Goren A, Gymrek M (2019). The impact of short tandem repeat variation on gene expression. Nat Genet.

[36] Fecto F, Yan J, Vemula SP, Liu E, Yang Y, Chen W (2011). SQSTM1 Mutations in familial and sporadic amyotrophic lateral sclerosis. JAMA Neurol.

[37] Rea SL, Majcher V, Searle MS, Layfield R (2014). SQSTM1 mutations–bridging Paget disease of bone and ALS/FTLD. Exp Cell Res.

[38] Al-Chalabi A, Andersen PM, Chioza B, Shaw C, Sham PC, Robberecht W (1998). Recessive amyotrophic lateral sclerosis families with the D90A SOD1 mutation share a common founder: evidence for a linked protective factor. Hum Mol Genet.

[39] Saeed M, Yang Y, Deng HX, Hung WY, Siddique N, Dellefave L, Gellera C, Andersen PM, Siddique T (2009). Age and founder effect of SOD1 A4V mutation causing ALS. Neurology.

[40] Pytte J, Flynn LL, Anderton RS, Mastaglia FL, Theunissen F, James I, et al. Disease-modifying effects of an SCAF4 structural variant in a predominantly SOD1 ALS cohort. Neurol Genet. 2020;6:e470. 10.1212/NXG.0000000000000470. 10.1212/NXG.0000000000000470 PMC7357414 32754644

[41] Gregersen LH, Mitter R, Ugalde AP, Nojima T, Proudfoot NJ, Agami R (2019). SCAF4 and SCAF8, mRNA anti-terminator proteins. Cell.

[42] Fliedner A, Kirchner P, Wiesener A, van de Beek I, Waisfisz Q, van Haelst M, Scott DA, Lalani SR, Rosenfeld JA, Azamian MS, Xia F, Dutra-Clarke M, Martinez-Agosto JA, Lee H, Noh GJ, Lippa N, Alkelai A, Aggarwal V, Agre KE, Gavrilova R, Mirzaa GM, Straussberg R, Cohen R, Horist B, Krishnamurthy V, McWalter K, Juusola J, Davis-Keppen L, Ohden L, van Slegtenhorst M, de Man SA, Ekici AB, Gregor A, van de Laar I, Zweier C, Nelson SF, Grody WW, Lee H, Deignan JL, Kang SH, Arboleda VA, Senaratne TN, Dorrani N, Dutra-Clarke MS, Kianmahd J, Hinkamp FL, Neustadt AM, Martinez-Agosto JA, Fogel BL, Quintero-Rivera F (2020). Variants in SCAF4 cause a neurodevelopmental disorder and are associated with impaired mRNA processing. Am J Hum Genet.

[43] Miller T, Cudkowicz M, Shaw PJ, Andersen PM, Atassi N, Bucelli RC, Genge A, Glass J, Ladha S, Ludolph AL, Maragakis NJ, McDermott CJ, Pestronk A, Ravits J, Salachas F, Trudell R, van Damme P, Zinman L, Bennett CF, Lane R, Sandrock A, Runz H, Graham D, Houshyar H, McCampbell A, Nestorov I, Chang I, McNeill M, Fanning L, Fradette S, Ferguson TA (2020). Phase 1–2 trial of antisense oligonucleotide tofersen for SOD1 ALS. N Engl J Med.

[44] Ze M, Lopez-Erauskin J, Baughn MW, Zhang O, Drenner K, Sun Y (2019). Premature polyadenylation-mediated loss of stathmin-2 is a hallmark of TDP-43-dependent neurodegeneration. Nat Neurosci.

[45] Klim JR, Williams LA, Limone F, San Juan IG, Davis-Dusenbery BN, Mordes DA, et al. ALS-implicated protein TDP-43 sustains levels of STMN2, a mediator of motor neuron growth and repair. Nat Neurosci. 2019;1. 10.1038/s41593-018-0300-4 PMC7153761 30643292

[46] Theunissen F, Anderton RS, Mastaglia FL, Flynn LL, Winter SJ, James I, et al. Novel STMN2 variant linked to amyotrophic lateral sclerosis risk and clinical phenotype. Front Aging Neurosci. 2021;13:127. 10.3389/fnagi.2021.658226 PMC8033025 33841129

[47] Prudencio M, Humphrey J, Pickles S, Brown A-L, Hill SE, Kachergus J, Shi J, Heckman MG, Spiegel MR, Cook C, Song Y, Yue M, Daughrity LM, Carlomagno Y, Jansen-West K, de Castro CF, DeTure M, Koga S, Wang YC, Sivakumar P, Bodo C, Candalija A, Talbot K, Selvaraj BT, Burr K, Chandran S, Newcombe J, Lashley T, Hubbard I, Catalano D, Kim D, Propp N, Fennessey S, Fagegaltier D, Phatnani H, Secrier M, Fisher EM, Oskarsson B, van Blitterswijk M, Rademakers R, Graff-Radford NR, Boeve BF, Knopman DS, Petersen RC, Josephs KA, Thompson EA, Raj T, Ward M, Dickson DW, Gendron TF, Fratta P, Petrucelli L, NYGC ALS Consortium (2020). Truncated stathmin-2 is a marker of TDP-43 pathology in frontotemporal dementia. J Clin Investig.

[48] Wang Q, Zhang Y, Wang M, Song W-M, Shen Q, McKenzie A, Choi I, Zhou X, Pan PY, Yue Z, Zhang B (2019). The landscape of multiscale transcriptomic networks and key regulators in Parkinson’s disease. Nat Commun.

[49] Van Eijk RPA, Nikolakopoulos S, Roes KCB, Kendall L, Han SS, Lavrov A, et al. Challenging the established order: innovating clinical trials for amyotrophic lateral sclerosis. Neurology. 2021. 10.1212/WNL.0000000000012545. 10.1212/WNL.0000000000012545 PMC8456357 34315786

[50] Roggenbuck J. C9orf72 and the care of the patient with ALS or FTD: Progress and recommendations after 10 years. Neurol Genet. 2021;7(1). 10.1212/NXG.0000000000000542 PMC7862089 33575483

[51] Nelson MR, Tipney H, Painter JL, Shen J, Nicoletti P, Shen Y, Floratos A, Sham PC, Li MJ, Wang J, Cardon LR, Whittaker JC, Sanseau P (2015). The support of human genetic evidence for approved drug indications. Nat Genet.

[52] Rotunno MS, Bosco DA (2013). An emerging role for misfolded wild-type SOD1 in sporadic ALS pathogenesis. Front Cell Neurosci.

[53] Hayashi Y, Homma K, Ichijo H (2016). SOD1 in neurotoxicity and its controversial roles in SOD1 mutation-negative ALS. Adv Biol Regul.

[54] Bennett CF, Kordasiewicz HB, Cleveland DW (2020). Antisense drugs make sense for neurological diseases. Ann Rev Pharmacol Toxicol.

[55] Petrov D, Mansfield C, Moussy A, Hermine O. ALS clinical trials review: 20 years of failure. Are we any closer to registering a new treatment? Front Aging Neurosci. 2017;9:68. 10.3389/fnagi.2017.00068 PMC5360725 28382000

[56] Wobst HJ, Mack KL, Brown DG, Brandon NJ, Shorter J (2020). The clinical trial landscape in amyotrophic lateral sclerosis—past, present, and future. Med Res Rev.

[57] Vejux A, Namsi A, Nury T, Moreau T, Lizard G. Biomarkers of amyotrophic lateral sclerosis: current status and interest of oxysterols and phytosterols. Front Mol Neurosci. 2018;11:12. 10.3389/fnmol.2018.00012 PMC5797798 29445325

[58] Khalil M, Teunissen CE, Otto M, Piehl F, Sormani MP, Gattringer T, Barro C, Kappos L, Comabella M, Fazekas F, Petzold A, Blennow K, Zetterberg H, Kuhle J (2018). Neurofilaments as biomarkers in neurological disorders. Nat Rev Neurol.

[59] Agah E, Saleh F, Moghaddam HS, Saghazadeh A, Tafakhori A, Rezaei N (2018). CSF and blood biomarkers in amyotrophic lateral sclerosis: protocol for a systematic review and meta-analysis. Syst Rev.

[60] Mitsumoto H, Saito T (2018). A prognostic biomarker in amyotrophic lateral sclerosis. Clin Neurol.

